# Addition of Clomiphene Citrate to a Low-Dose Gonadotropin-Releasing Hormone (GnRH)-Antagonist Protocol in Poor Responders: A Prospective Cohort Study

**DOI:** 10.7759/cureus.108079

**Published:** 2026-05-01

**Authors:** Olga Triantafyllidou, Evangelia Panagodimou, Stamatia Chasiakou, Angelos Gkontelos, Konstantinos Karkalemis, Maria Simopoulou, Despina Tzanakaki, Nikolaos Syggelos, Theodoros Kalampokas, Panagiotis Vakas, Alexandros Lazaridis, Fotios Vlahos, Panagiotis Christopoulos, Nikolaos Vlahos

**Affiliations:** 1 Second Department of Obstetrics and Gynaecology, Aretaieio University Hospital Medical School, National and Kapodistrian University of Athens, Athens, GRC; 2 Department of Obstetrics and Gynaecology, IASO General Hospital, Athens, GRC; 3 Department of Family Medicine, Hippokration General Hospital, Athens, GRC; 4 Department of Primary Education, University of Crete, Rethymno, GRC; 5 Department of Physiology, Aretaieio University Hospital Medical School, National and Kapodistrian University of Athens, Athens, GRC; 6 IVF Unit, Second Department of Obstetrics and Gynaecology, Aretaieio University Hospital Medical School, National and Kapodistrian University of Athens, Athens, GRC; 7 School of Medicine, University of Milano Bicocca, Milan, ITA

**Keywords:** add-on, clomiphene citrate, gnrh antagonist protocol, in vitro fertilization (ivf), poor responders, retrospective cohort study

## Abstract

Objective: To evaluate whether the addition of clomiphene citrate (CC) to a low-dose recombinant follicle-stimulating hormone (FSH) (r-FSH) gonadotropin-releasing hormone (GnRH)-antagonist protocol improves clinical outcomes in women with poor ovarian response (POR) compared with a standard high-dose antagonist regimen.

Methods: This prospective cohort study included 31 women with POR according to the Bologna criteria who underwent two sequential in vitro fertilization (IVF)/intracytoplasmic sperm injection (ICSI) stimulation cycles: a standard-dose r-FSH protocol (300 IU/day), followed by a low-dose r-FSH protocol (150 IU/day) combined with CC (100 mg/day, cycle days 3-7). Each patient served as her own control. The primary outcome was the ongoing pregnancy rate. Secondary outcomes included follicle number, metaphase II (MII) oocytes, embryos, fertilization rate, biochemical and clinical pregnancy, and miscarriage. Statistical analyses included paired t-tests, Wilcoxon signed-rank tests, and Fisher's exact tests.

Results: The number of follicles ≥ 17 mm and the number of mature oocytes retrieved were comparable between the standard-dose and CC/low-dose protocols (3.00 vs 2.90 follicles, p=0.665; 2.19 vs 2.06 MII oocytes, p = 0.462). Fertilization rates (27/68 (39.7%) vs 35/64 (54.7%), p = 0.116), total embryos (27 (0.87) vs 35 (1.13), p = 0.101), transferred embryos, and biochemical or clinical pregnancy rates did not differ significantly between protocols. No ongoing pregnancies occurred in the standard group, while three (3/31) were achieved after CC/low-dose stimulation (0% vs 9.7%, p = 0.238).

Conclusions: In women with POR, adding CC to a low-dose GnRH-antagonist protocol results in comparable ovarian response and pregnancy outcomes to a standard high-dose regimen, with the potential advantages of lower gonadotropin use and reduced cost. CC-based mild stimulation represents a viable alternative following an unsuccessful high-dose cycle.

## Introduction

Poor ovarian response (POR) to controlled ovarian stimulation (COS) continues to present a major clinical dilemma in reproductive medicine, despite considerable progress in assisted reproductive technologies (ART). POR is defined as a diminished ovarian response and a suboptimal follicular output following a standard protocol of ovarian stimulation. This leads to a reduced number of oocytes, a higher incidence of cycle cancellation, and decreased live birth rates. Consequently, this condition is a significant source of psychological distress for affected patients [1]. Large variations in defining POR have resulted in heterogeneous populations of patients in various clinical trials [2]. This makes it difficult to come to definite and safe conclusions. Hence, no particular treatment option is considered better than others [3].

The first attempt to establish a definition for POR was the publication of the European Society of Human Reproduction and Embryology (ESHRE)-approved Bologna criteria [4], according to which, a patient is considered a poor responder if two out of the following three are met: (i) advanced maternal age (≥ 40 years) or any other risk factor for POR; (ii) a previous POR (≤ 3 oocytes with a conventional stimulation protocol); (iii) an abnormal ovarian reserve test (i.e., antral follicle count (AFC) < 5-7 follicles or anti-Müllerian hormone (AMH) < 0.5-1.1 ng/mL). The prevalence of poor responders according to these criteria was estimated between 8.1-39.1% of women undergoing IVF treatments [5]. The Bologna criteria, however, do not provide any guidance on patient counselling or clinical management of poor responders [6]. The Patient-Oriented Strategies Encompassing IndividualizeD Oocyte Number (POSEIDON) criteria, introduced in 2016, were designed to address the limitations of the earlier Bologna criteria. This stratification has been developed to create a more nuanced, patient-centric classification that not only predicts the oocyte yield but also provides patient-specific therapeutic strategies. Patients are classified in four groups based on various qualitative and quantitative characteristics, such as age, AFC, AMH, and previous ovarian response [7]. Despite etiological stratification, prognostic improvement, and promotion of personal treatment, the POSEIDON criteria seem to add complexity to clinical practice, focusing on the oocyte number rather than the final results.

A review of the various strategies and modified COS protocols, which have been developed to optimize the management of PORs, reveals a lack of specific evidence supporting the efficacy of one approach over another [8,9]. Despite the high doses of gonadotropins, patients with POR often yield a limited number of oocytes and consequently lower pregnancy rates [10]. Mild ovarian stimulation has emerged as an alternative to conventional high-dose ovarian stimulation for women with diminished ovarian reserve or poor responders [11]. Mild stimulation involves the administration of low-dose gonadotropins as monotherapy or in combination with clomiphene citrate (CC), aromatase inhibitors, gonadotropin-releasing hormone (GnRH) antagonists, and late follicular phase human chorionic gonadotropin/luteinizing hormone (hCG/LH). In fact, mild stimulation protocols with either low-dose gonadotropins (≤ 150 IU/day) alone or in combination with an oral agent resulted in comparable clinical pregnancy rates to conventional high-dose gonadotropin protocols [12]. Mild stimulation has also been associated with reduced gonadotropin dosages and lower costs [12,13].

CC, a selective estrogen receptor modulator, has been used in mild stimulation protocols due to its ability to stimulate endogenous pituitary gonadotropins and increase granulosa cell sensitivity to these hormones [14,15]. CC was initially introduced for ovarian stimulation in poor responders in 2004 [16]. Since the introduction of the Bologna criteria, several clinical trials and meta-analyses have evaluated the impact of different CC dosages, with and without gonadotropins, on ovarian stimulation outcomes in poor responders.

This study aims to evaluate whether the addition of CC to a low-dose gonadotropin regimen (150 IU/day) within a GnRH antagonist protocol is non-inferior to the standard-dose GnRH antagonist regimen in terms of reproductive outcome, specifically ongoing clinical pregnancy rate in women with POR.

## Materials and methods

This prospective cohort study was approved by the ethics committee of the Aretaieio University Hospital of Athens (protocol N. 613/14-10-2024). The study was also reported at the clinical trials registry (clinicalTrials.gov) with registration number NCT06778733. The study was conducted at the Assisted Reproductive Unit of the Second Department of Obstetrics and Gynecology, "Aretaieio" Hospital, National and Kapodistrian University of Athens, Greece, from January 2022 to April 2025, and included women with POR according to the Bologna criteria [4]. At least two out of the following criteria had to be met: patient's age over 40 years, AMH less than 1.1 ng/mL, at least one previous cycle of ovarian stimulation with three or fewer oocytes, or a cancelled attempt due to poor response.

The exclusion criteria for participation in our study were severe male factor infertility, abnormal endometrial cavity according to transvaginal ultrasound and hysterosalpingography or hysterosalpingo-foam sonography (HyFoSy), adenomyosis, day three follicle-stimulating hormone (FSH) higher than 17 IU/l, and estradiol level at day three higher than 70 pg/mL. In addition, patients were instructed to quit smoking for at least two months before enrollment, and patients with endocrine disorders (including diabetes mellitus) or obesity (BMI > 30.0) were also excluded from the study. Our study did not include preimplantation genetic screening.

**Figure 1:**
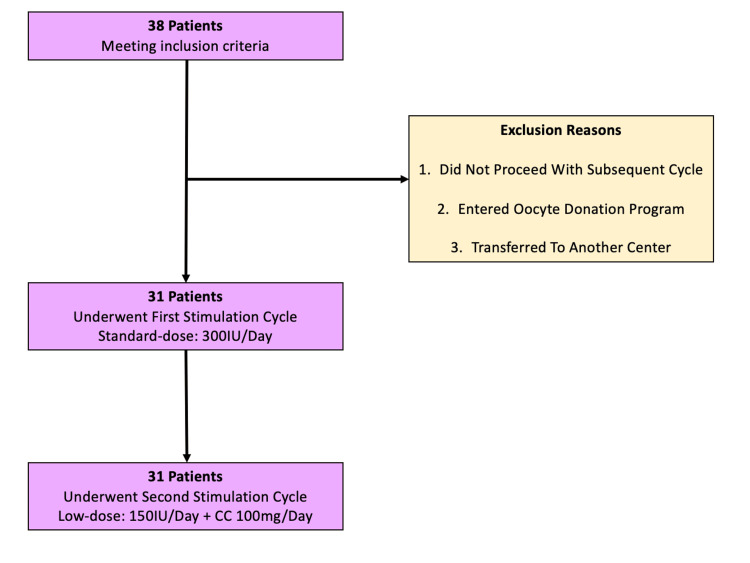
Patient flow diagram

A patient flow diagram is provided in Figure 1. Initially, 38 patients who met the inclusion requirements were enrolled in the study. Of these, three decided to continue their stimulation at another assisted reproduction centre, while three women eventually opted for oocyte donation. One patient did not proceed with a subsequent stimulation cycle. Finally, the study included 31 patients who underwent a standard-dose (300 IU/day) gonadotropin/GnRH antagonist ovarian stimulation protocol, in accordance with the ESHRE 2025 guideline for ovarian stimulation. One to three months later, the same group underwent a second stimulation cycle with low-dose gonadotropins (150 IU/day) until the day of hCG administration, with the addition of CC (100 mg/day) from day three to day seven of the menstrual cycle. Every patient underwent only one stimulation protocol in each group. Written informed consent was obtained from all patients.

Treatment schedule

Initially, all participants were assigned to the standard-dose short GnRH antagonist protocol. Serum AMH levels were measured before initiating COS. Estradiol and FSH levels were measured on days two to three of the cycle, and stimulation began on the third day with the administration of 300 IU of recombinant human FSH (r-FSH-alfa) (Gonal-f; Merck Serono Hellas AE, Athens, Greece) after having performed a transvaginal ultrasound, provided that serum FSH levels were under 17 mIU/mL and estradiol under 70 pg/mL (r-FSH group). All patients underwent routine monitoring with transvaginal ultrasound and serum estradiol measurements. The GnRH antagonist cetrorelix acetate, at a dose of 0.25 mg/day (Cetrotide; Merck Serono Hellas AE, Athens, Greece), was administered as soon as a dominant follicle reached 14 mm in diameter. Oocyte retrieval (OPU) was performed 34-36 hours after hCG administration (Ovitrelle; Merck Serono Hellas AE), followed by sequential in vitro fertilization (IVF)/intracytoplasmic sperm injection and embryo transfer on day three after OPU. Each embryo was evaluated at the cleavage stage, based on the grading system suggested by Veeck [17]. Micronized progesterone (Utrogestan; 200 mg/4 daily vaginally, Angelini Pharma ABEE, Attica, Greece) was administered for luteal phase support. Serum β-hCG levels were measured 12 days after embryo transfer, and clinical pregnancy was confirmed by transvaginal ultrasound 14 days later. After a negative result, patients were assigned to the CC/r-FSH group, undergoing a low-dose ovarian stimulation protocol with r-FSH (150 IU/day from cycle day three) in combination with CC (100 mg/day for days 3-7). The endometrial thickness and morphology were evaluated in each visit. For patients with an endometrial thickness less than 8 mm on the day of triggering, a freeze-all strategy was employed. Embryo transfer was performed in a subsequent hormonally prepared cycle, using oral estradiol valerate (2 mg, three times daily) (Cyclacur, Bayer, Berlin, Germany). The period between the two stimulations ranged from one to three months. The treatment schedule was the same, and an ongoing pregnancy was confirmed at 12 weeks by ultrasound. The follow-up period for all patients was 12 gestational weeks. Beyond that point, patients were treated by clinicians of their choice outside our centre. In both protocols, during the follow-up period, the clinicians were aware of the treatment schedule given to each patient.

Outcome measures

The primary outcome measure was ongoing pregnancy rates. Secondary outcomes included the number of follicles and mature oocytes, fertilization rate, and total number of embryos in each group. Furthermore, we included biochemical pregnancy rates and miscarriage as secondary outcomes. Complete follow-up to live birth was not consistently available due to the continuation of obstetrical care across multiple private practices and institutions, besides our assisted reproductive unit.

Statistical analysis

Analysis was conducted with Statistical Product and Service Solutions (SPSS, version 26; IBM SPSS Statistics for Windows, Armonk, NY). We performed an a priori power calculation test in order to calculate the minimum sample size needed, in matched/dependent pairs, two-tailed, given a type a error probability of 0.05 and power (type 1-β error probability) of 0.8. In order to detect a large effect size (d) of 0.8, the sample size required was calculated at 15 pairs (G-power 3.1). In our study, we included 31 patients, which is considered more than adequate, according to a priori power calculation. Continuous data are presented as mean values and standard error of the mean and were compared using the Wilcoxon signed-rank test or paired Samples t-test, where appropriate, depending on the normality of the data. Categorical variables were analysed using Fisher's exact test. A p-value < 0.05 was considered statistically significant.

## Results

The study included 31 women who underwent two subsequent cycles of ovarian stimulation. The first one was the standard gonadotropin antagonist protocol (r-FSH group) as described above, and the next cycle was the low-dose r-FSH protocol with the addition of 100 mg of CC (CC/r-FSH group). Each patient served as her own control for the two treatments. Baseline characteristics of the groups are shown in Table 1.

**Table 1:** Clinical and hormonal data of the two groups FSH: follicle-stimulating hormone; CC: clomiphene citrate; AMH: anti-Müllerian hormone; W: Wilcoxon signed-rank test; P: paired Samples t-test

Variables	r-FSH group, n=31	CC/r-FSH group, n=31	Test statistics	p-value
Age (years)	40.16 ± 0.63	40.52 ± 0.63	-3.317 (W)	0.001
Day 3 FSH ± SE	9.56 ± 0.43	10.01 ± 0.36	-1.224 (P)	0.23
Day 3 estradiol ± SE	49.88 ± 2.33	46.44 ± 1.91	-1.911 (W)	0.056
AMH ± SE	0.65 ± 0.05	0.62 ± 0.48	3.639 (P)	0.001

The mean age of women included in our study was 40.16 and 40.52 years for the r-FSH and CC/r-FSH groups, respectively. Although the mean age was statistically significantly higher in the CC/r-FSH group (p = 0.001), its clinical relevance may be limited due to the small effect size. Patients' characteristics, including baseline FSH (9.56 vs 10.01, t = -1.224, p = 0.230) and estradiol levels (49.88 vs 46.44, z = -1.911, p = 0.067), were similar between the two groups (Table 1). Interestingly, there was a statistically significant difference in AMH levels between the two groups (0.65 vs 0.62, p = 0.001), with slightly lower AMH levels in the second group. Stimulation characteristics and outcomes are summarized in Table 2.

**Table 2:** Stimulation results (clinical, hormonal, and embryologic data) of the two groups W: Wilcoxon signed-rank test; F: Fisher's exact test

Variables	r-FSH group, n=31	CC/r-FSH group, n=31	p-value	Statistical test
Number of follicles ± SE	3.00 ± 0.27	2.90 ± 0.21	0.665	-0.433 (W)
Number of oocytes ± SE	2.19 ± 0.22	2.06 ± 0.21	0.462	-0.736 (W)
Fertilization rate	27/68 (39.7%)	35/64 (54.7%)	0.116	F
Number of embryos ± SE	0.87 ± 0.14 (n=27)	1.13 ± 0.14 (n=35)	0.101	-1.641 (W)
Positive β-hCG	5/31 (16.1%)	7/31 (22.6%)	0.749	F
Biochemical pregnancy rates	2/31 (6.5%)	2/31 (6.5%)	1	F
Miscarriage pregnancy rates	3/31 (9.7%)	2/31 (6.5%)	1	F
Clinical pregnancy rate	3/31 (9.7%)	5/31 (16.1%)	0.707	F
Ongoing pregnancy rates	0/31 (0.00%)	3/31 (9.7%)	0.238	F

The number of follicles >17 mm in diameter measured by transvaginal ultrasound on the day of triggering was similar between the two groups (3.00 vs 2.90, z = -4.33, p = 0.665). The number of metaphase II (MII) oocytes retrieved was similar in both groups as well (2.19 vs 2.06, t = -0.736, p = 0.462). Subsequently, there were no statistically significant differences between the two groups regarding the total number of embryos (0.87 vs 1.13, z = -1.641, p = 0.101) or the fertilization rates (54.6% in CC/r-FSH group vs 39.7% in r-FSH group, p = 0.116). There was no statistically significant difference in the number of transferred embryos (27/31 vs 35/31, p = 0.101). No statistically significant differences were detected in biochemical pregnancies (6.5% (2 out of 31) vs 6.5% (2 out of 31), p = 1.000) or miscarriages (9.7% (3 out of 31) vs 6.5% (2 out of 31), p = 1.000) before and after the supplementation of 100 mg of CC, respectively. Two clinical pregnancies were confirmed in each group, but they resulted in miscarriages (p = 0.707). Similarly, no significant differences were detected in the total number of pregnancies, as there were five positive β-hCG tests in the r-FSH group, as opposed to seven in the CC/r-FSH group (p = 0.749).

Finally, focusing on the primary outcome (ongoing pregnancies), there were none in the r-FSH group, whereas in the CC/r-FSH group, there were three confirmed ongoing pregnancies out of the 31 embryo transfers (9.7%). However, this difference did not reach statistical significance (p = 0.238).

## Discussion

This prospective cohort study evaluated the efficiency of the addition of CC to a low-dose gonadotropin protocol, compared to conventional standard-dose GnRH-antagonist protocols, among patients with a POR as defined by the Bologna criteria. In our study, the mild stimulation protocol demonstrated comparable results to high-dose gonadotropin regimens. Our findings suggest that there is no statistically significant difference between the two protocols regarding the ongoing or clinical pregnancy rates, as well as miscarriage rates, the number of developed follicles, oocytes retrieved, or embryos. These results contribute to the ongoing debate regarding the optimal stimulation treatment for PORs.

POR to stimulation with high-dose gonadotropins is observed in 9%-24% of ovarian stimulation cycles and is associated with significantly reduced oocyte yield and pregnancy rates [18]. This evidence highlighted the need for alternative therapeutic strategies. CC is one of the most commonly used pharmacological agents for poor responders. The positive effect of CC in combination with low doses of gonadotropins appears to be confirmed in patients who showed an unexpectedly low response to an initial standard stimulation protocol. One of the first studies evaluating the combination of CC and gonadotropins was published in 1976 [19]. Since then, a lot of studies have reported that combined use of CC and gonadotropins is an effective alternative choice for COS in poor responders [8,16,20,21].

Consensus with existing literature: non-superiority of specific regimens

Our results are in agreement with a large amount of evidence suggesting that there is no significant difference in any of the ovarian stimulation protocols in PORs. This is supported by several cohort and randomized controlled trials (RCTs) and meta-analyses. Pilehvari et al. compared high-dose gonadotropins (35 patients) to a regimen of CC plus low-dose human menopausal gonadotropin (hMG) (42 patients), and there was no significant difference in the number of oocytes retrieved or pregnancy rates [22]. Further supporting this, Moffat et al., in a multi-arm randomized trial, found no statistically significant difference in oocyte yield between groups receiving different combinations of gonadotropin doses and CC [23]. They divided participants into four groups according to the treatment protocol received (Group A: 450 IU/day gonadotropins plus 100 mg CC, Group B: 150 IU/day gonadotropins plus 100 mg CC, Group C: 450 IU/day gonadotropins, and Group D: 150 IU/day gonadotropins). There was no statistically significant difference between the groups in the number of oocytes retrieved; however, the number of blastocysts was significantly different in favor of Group B (150 IU/day gonadotropins plus 100 mg CC), hinting at a potential qualitative benefit without a quantitative increase in oocytes [23]. These results indicate the potential protective role of CC against the over-suppression of the hypothalamic-pituitary axis and the potential depletion of ovarian reserve with the use of high doses of gonadotropins [21]. This evidence was summarised in 2017 in two meta-analyses, which included 27 RCTs and showed that reproductive outcomes were similar in different stimulation protocols in women with POR [24,25].

The trend towards comparable pregnancy rates, even in the presence of variable oocyte yields, is crucial. For instance, Siristatidis et al. and Revelli et al. both reported a significantly higher number of oocytes retrieved using high-dose long GnRH agonist protocols compared to mild CC-containing long protocols [8,20]. Siristatidis et al. in 58 poor responders reported a significantly higher mean number of cumulus oocyte complexes retrieved in the high-dose group compared to the low-dose group with CC (1 (0-4) vs 3 (0-8.4), p < 0.001), whereas live birth (9.1% vs 12%), clinical pregnancy (12.1% vs 20%), and miscarriage rates (40% vs 40%) were similar in the two groups [20]. The same results were concluded in an RCT conducted by Revelli et al. three years earlier [8], where, despite more favourable results in terms of number of oocytes and embryos in the high-dose long protocol group, they obtained comparable implantation rates, clinical pregnancy rates, and ongoing pregnancy rates in both groups of patients ("mild" CC/FSH vs "long" protocol). In the context of diminished ovarian reserve, the effect of high doses of gonadotropins is thought to interfere with the completion of meiosis, leading to poor-quality embryos despite the increased number of oocytes retrieved in women with already extremely low oocyte quality [26]. Furthermore, our previously published study supported that the addition of 100 mg CC to the high-dose stimulation protocol in women with POR could lead to better stimulation outcomes, but could not affect the pregnancy rates [27]. Similarly, a recent study by Liu et al., including 294 patients [28], showed that adding CC to a GnRH antagonist protocol can result in an increase in follicle-to-oocyte index. However, multivariate regression analysis showed that cumulative ongoing pregnancy was not significantly different in the two groups.

Clinical pregnancy rates and, especially, ongoing pregnancy rates are significant outcomes for measuring the efficacy of ART protocols. Despite the higher oocyte yield achieved with the high-dose protocol for poor responders, the pregnancy and live birth rates for both protocols were comparable in the studies mentioned earlier. The findings of our study are in support of the trending literature that there are no significant differences in clinical pregnancy rates between high-dose gonadotropin and low-dose plus CC protocols. Although there were three ongoing pregnancies in the low-dose group, the difference was not statistically significant. The concept of "less is more" is emphasized in the work of Weghofer et al. [29]. The authors showed in their study that women with POR can benefit from a minimal stimulation approach using a low dose of gonadotropins with similar pregnancy outcomes compared to the standard approach (10% vs 10.7% per embryo transfer). The authors emphasized this as it is "cost-effective" [29]. This is further highlighted in the systematic review of Kamath et al., which included 3,599 participants from 22 studies. The authors showed that there was no significant difference in live birth and pregnancy rates between groups, with and without the use of CC in standard protocols of gonadotropins, resulting in a significant reduction of total dose and duration of gonadotropin treatment [24].

Evidence supporting the efficacy of CC-containing protocols

On the other hand, there are studies that support a beneficial effect from adding CC in GnRH antagonist protocols in poor responders [30,31]. In 2018, Ochin et al. showed a significantly higher number of oocytes retrieved (7.26 vs 5.98; p = 0.03) and a better cumulative pregnancy rate (9.2% vs 51%; p < 0001) in the CC/low-dose GnRh antagonist protocol [21]. The authors contributed these results to better bidirectional signalling between granulosa cells and oocytes and, therefore, to improve the quality of oocytes in the CC group. Moreover, their results suggest that CC could have a protective effect against over-suppression of hypothalamic-pituitary function and possible ovarian reserve depletion due to aggressive stimulation with high doses of gonadotropins. To avoid the negative effect of CC on endometrial thickness and embryo implantation [32,33], Ochin et al. used a freeze-all strategy. In our study, endometrial thickness and morphology were assessed on the day of triggering in all patients. In patients with unfavorable endometrium, a freeze-all strategy was employed (8/31 patients), and embryo transfer was performed in a subsequent cycle. Similarly, Liang et al. included a total number of 2724 cycles and demonstrated that the addition of CC resulted in higher cumulative pregnancy rates (39.74% vs 68.21%, p < 0.001) in poor responders [34].

It has to be mentioned that, despite the lack of statistical significance in outcomes in our study, the group receiving the CC/r-FSH protocol had a baseline profile of slightly older age (40.52 vs 40.16 years, p = 0.001) and lower AMH levels (0.65 vs 0.62, p = 0.001), suggesting a population with a potentially worse prognosis. Although these differences were statistically significant, due to the small size of our study, we strongly believe that their clinical impact is likely minimal but could be considered when interpreting results.

Low-dose gonadotropin protocols in combination with CC are generally considered more cost-effective than high-dose gonadotropin protocols. CC is an inexpensive oral medication, and its combination with lower doses of gonadotropins reduces the overall cost of IVF. A study by Satwik et al. demonstrated that the addition of CC led to a 28-40% reduction in the total cost of stimulation, by reducing the required gonadotropin dose [35]. This is further supported by a meta-analysis from Zhang et al. in 2020. The authors mentioned that the lower total gonadotropin dosage makes CC protocols an alternative treatment for POR and the most economical option [36].

The American Society for Reproductive Medicine (ASRM) has proposed the use of mild ovarian stimulation protocols in poor responders [12]. However, the ESHRE guidelines on COS report that there is not enough data to suggest the use of a concrete dose of gonadotropin in POR (strong recommendation), leaving the final decision to the clinicians [37]. There is no evidence to support the routine administration of CC in COS protocols for unselected patients [38]. The choice of protocol for poor responders should be individualized, taking into consideration the previous response to stimulation and the number of IVF cycles. High-dose gonadotropins may be considered as a first-line treatment in patients where the primary goal is to maximize the oocyte yield. However, this approach does not necessarily translate into higher clinical or ongoing pregnancy rates. On the other hand, low-dose gonadotropin protocols combined with CC offer a cost-effective alternative with similar clinical outcomes. Consequently, the addition of CC to the low-dose protocol in POR could be a useful alternative regimen in case of a negative result of a previous high-dose stimulation. Similar pregnancy, miscarriage, and ongoing pregnancy rates indicate that oocyte quality may be more important than quantity for successful outcomes in poor responders.

The findings of our study should be interpreted with caution, taking into account its main limitations, which are the sample size, the study design (a prospective cohort study and not a randomized controlled trial), and the fact that the data were collected from patients of only one institution. Within these constraints, no statistically significant differences were observed between the two protocols; thus, these results are consistent with the non-inferiority of the low-dose GnRH antagonist protocol combined with CC. Moreover, our study compared a low-dose GnRH antagonist protocol plus CC with a standard-dose protocol without rLH supplementation. Clinical evidence suggests a beneficial effect of rLH supplementation in POR, specifically in women aged > 35 years [6,39-41]. Another limitation of our study was the day of embryo transfer. We performed embryo transfer at the cleavage stage instead of the blastocyst stage. Moreover, it is to be noted that the follow-up in this study was only extended to the 12th week of pregnancy, and the care was handed over to the clinicians of the patients' choice. On the other hand, one of the major strengths of the study was that it followed a paired study design in which the same patient group was exposed to both interventions. This design is used to control for all confounding variables.

## Conclusions

Both high-dose and low-dose gonadotropin protocols, in addition to CC, are effective treatment options for women diagnosed with POR. The high-dose protocol could result in a higher number of oocytes retrieved, but this does not necessarily lead to improved pregnancy or live birth rates. On the other hand, this current study has shown similar outcomes in terms of ovarian stimulation and clinical pregnancy rates using the low-dose protocol along with CC, with the additional benefit of reduced costs and lower amount of gonadotropins. Further studies should aim to refine and optimize patient selection criteria to maximize outcomes with each of these protocols, ensuring that poor responders are receiving the best and most effective treatment available. The results of this study suggest that the addition of CC to a low-dose gonadotropin regimen is an effective alternative to the traditional high-dose gonadotropin approach in PORs. One of the main advantages of the low-dose gonadotropin plus CC protocol is its cost-effectiveness, making ART more accessible to a broader range of patients.
